# Characterizing Pain in Peripheral Nerve Tumors: Interim Results from a Prospective Bicenter Cohort

**DOI:** 10.3390/cancers18101517

**Published:** 2026-05-08

**Authors:** Nadja Grübel, Anne-Kathrin Uerschels, Karsten Wrede, Nora F. Dengler, Benjamin Mayer, Christian Rainer Wirtz, Maria Teresa Pedro

**Affiliations:** 1 Peripheral Nerve Unit, Department of Neurosurgery, University of Ulm, Lindenallee 2, 89312 Günzburg, Germanymaria-teresa.pedro@uni-ulm.de (M.T.P.); 2 Department of Neurosurgery, University of Essen, Hufelandstraße 55, 45147 Essen, Germany; 3 Department of Neurosurgery, Johannes-Müller-Straße 7, 56068 Koblenz, Germany; 4 Department of Neurosurgery, Helios Hospital Bad Saarow, Pieskower Str. 33, 15526 Bad Saarow, Germany; 5 Institute of Epidemiology and Medical Biometry, Ulm University, 89075 Ulm, Germany

**Keywords:** pain assessment, neuropathic pain, peripheral nerve tumor, PainDETECT, schwannoma, MPNST

## Abstract

**Background/Objectives**: This prospective study used a standardized preoperative assessment to characterize the full spectrum of pain in patients with peripheral nerve tumors, addressing the lack of structured pain phenotyping in this population. **Methods**: Between June 2024 and October 2025, preoperative pain symptoms were assessed in 91 patients, representing 16% of the Peripheral Nerve Tumor Registry (PNTR) cohort, using the PainDETECT questionnaire (0–38) at two centers: University Hospital Ulm, Günzburg (*n* = 72), and University Hospital Essen (*n* = 19). **Results**: Ninety-one patients (61.5% male; mean age, 49 years) were included. Most tumors were located in the lower extremity (54.9%). PainDETECT scores were ≤12 in 51.6%, 13–18 in 24.1%, and ≥19 in 21.9% of patients. Malignant tumors (6.5%) had the highest mean score (17), but benign tumors also showed a relevant pain burden (mean 11.9). Hybrid peripheral nerve sheath tumors and neurofibromas had numerically higher mean scores than schwannomas, although the difference was not statistically significant. Pain severity was not associated with tumor size, depth, or affected nerve. The most common sensory features were electric-shock-like and pressure-related pain, as reflected by a positive Tinel sign in 86.8% of patients. The most frequent pain pattern was intermittent attacks with pain-free intervals (46%). **Conclusions**: These interim results indicate that pain is common even in benign peripheral nerve tumors, challenging the assumption that these lesions are often asymptomatic. Malignant tumors showed the highest pain scores. The heterogeneous pain phenotypes highlight the need for individualized assessment and management. Expansion to a multicenter evaluation within the framework of the PNTR is planned.

## Simple Summary

This prospective bicenter study systematically evaluated pain in 91 patients with peripheral nerve tumors using the PainDETECT questionnaire. The mean age was 49 years, and most tumors were located in the lower extremities. More than half of the patients had PainDETECT scores ≤ 12, whereas approximately one-fifth had scores ≥ 19, suggesting likely neuropathic pain. Malignant tumors showed the highest mean pain scores, but benign tumors also demonstrated a relevant pain burden, challenging the assumption that they are usually painless. Hybrid nerve sheath tumors and neurofibromas showed numerically higher scores than schwannomas, although these differences were not statistically significant. Pain scores were not significantly associated with tumor size, depth, or affected nerve. Common symptoms included electrifying and pressure-related pain, and nearly 87% of patients had a positive Tinel sign. Overall, pain phenotypes in peripheral nerve tumors were heterogeneous, underscoring the need for individualized assessment and management.

## 1. Introduction

Pain is among the most frequent presenting symptoms of peripheral nerve tumors (PNT) and should be systematically considered during diagnostic work-up and surgical planning [1]. Patients commonly report pain in benign entities—including schwannomas and neurofibromas—while some lesions remain asymptomatic; clinical signs such as a positive Tinel sign are frequent [2,3]. In schwannomatosis, chronic pain is highly prevalent and often difficult to treat [4,5,6]. Malignant peripheral nerve sheath tumors (MPNST) mostly present with severe pain, yet new or progressively worsening pain together with tumor growth constitutes a critical red flag for malignancy [7,8]. Pain is also a major driver of impaired health-related quality-of-life in this population [9,10]. Surgical resection of benign tumors is frequently associated with meaningful pain relief and satisfactory functional outcomes, supporting timely intervention in selected cases with substantial pain burden [10,11]. Together, the heterogeneity of pain presentations and neuropathic features across PNT subtypes supports prospective, structured assessment of the full pain spectrum to reduce diagnostic delays and guide patient-centered management [1,2,4,9,10].

### 1.1. Preliminary Clinical PNTR Findings

In an interim analysis of clinical data from the multicenter Peripheral Nerve Tumor Registry (PNTR) [12], pain was the most frequent presenting symptom among 280 patients with available 3-month follow-up data. Preoperatively, pain was routinely differentiated into rest pain and load-/activity-related pain. Postoperatively, we observed a significant reduction in overall pain (*p* < 0.001, McNemar test). Immediately after surgery, neurological deficits (rest and load pain, motor, and sensory deficits) were transiently more frequent, consistent with early postoperative effects; however, these deficits decreased over time, with the greatest improvement observed for pain. These registry signals motivated the present, more detailed, symptom-focused subanalysis in a surgically treated cohort. These Findings are therefore provided for context and should be interpreted separately from the primary results of this study.

### 1.2. Rationale for the Prospective Pain Sub-Study

These registry observations underscored the need for a prospective, structured characterization of pain phenotypes in peripheral nerve tumors. We therefore implemented a standardized preoperative and follow-up protocol. This sub-study was designed to capture the full spectrum of pain features, including intensity, quality, distribution, temporal pattern, and neuropathic characteristics, as well as their postoperative evolution, while aligning patient-reported outcomes with histology, imaging, and surgical variables.

## 2. Materials and Methods

This prospective PNTR sub-study was conducted at two neurosurgical centers in Germany: the Department of Neurosurgery, University Hospital Essen (*n* = 19), and the Section for Peripheral Nerve Surgery, Department of Neurosurgery, BKH Günzburg, Ulm University (*n* = 72). All patients provided written informed consent. The study followed institutional guidelines and was approved by the local ethics committees of both centers, with overarching approval by the Ethics Committee of Ulm University for registry management (No. 249/17). Data extracted from the clinical registry and operative records included patient characteristics (demographics and comorbidities); tumor location; histopathological diagnosis; surgical management (biopsy vs. gross-total/complete resection); and clinical symptoms/signs, including motor deficits (paresis), sensory deficits, and, based on structured history, pain at rest and load-/activity-related pain. The Tinel–Hoffman (Tinel’s) sign was assessed systematically. Data on comorbidities and medication were available for 73 of 91 patients (80.2%). In patients with multiple tumors, the most recently operated tumor for which a prospective PainDETECT assessment was available was defined as the index lesion. Previous tumor resections were documented retrospectively from medical records but were not included as separate tumor-specific pain assessments. Each patient was included only once in the present analysis. This cohort only included patients with peripheral nerve tumors or associated lesions; patients with purely spinal manifestations were excluded. Patients were categorized into four groups according to final histopathology: benign PNSTs (Group 1), malignant tumors (Group 2), rare/other peripheral nerve-associated tumor entities (Group 3), and dropout lesions (Group 4). Group 4 comprised cases in which a peripheral nerve tumor was suspected preoperatively due to close nerve contact on imaging and/or intraoperative findings, but histopathology ultimately revealed non-PNST tissue, such as non-neoplastic soft tissue or other lesions. This group was retained as a clinically meaningful comparison cohort because these lesions can mimic PNSTs through their close relationship to a nerve and therefore represent a relevant control group for evaluating symptom patterns and diagnostic features. This approach reflects the real-world diagnostic setting in which the final distinction between true peripheral nerve sheath tumors and nerve-adjacent tumor mimics is often only possible after histopathological confirmation. A further mechanistic distinction between extrinsic nerve compression, displacement, stretching, intraneural infiltration, or nerve destruction was not required for inclusion in this prospective interim analysis.

### 2.1. Study Protocol

Preoperatively, validated patient-reported outcome measures were used to assess pain. These included the PainDETECT questionnaire, which quantifies pain characteristics to estimate the likelihood of neuropathic pain and ranges from 0 to 38, with higher scores indicating greater pain burden. The overall score refers to the final summed PainDETECT score, including the pain course pattern and radiation items, whereas the end score refers to the summed symptom score without these additional modifiers [13,14]. Health-related quality of life was assessed using the five-level EuroQoL-5D-5L (EQ-5D-5L), including the dimension for pain and discomfort, together with the EuroQoL visual analog scale (EQ-VAS) [15]. Sleep quality was measured with the Pittsburgh Sleep Quality Index (PSQI) [16]. Follow-up consisted of a clinical examination and magnetic resonance imaging (MRI) surveillance at 3–6 months postoperatively, at which time all three questionnaires were re-administered.

This interim analysis was conducted after the first year of recruitment for prespecified descriptive and clinical purposes. Based on clinical observations and questionnaire review indicating higher-than-expected pain intensities, including in benign peripheral nerve tumors, the analysis aimed to provide an early characterization of preoperative pain phenotypes, assess their clinical relevance, and identify potential subgroups with higher pain burden to inform refinement of the ongoing PNTR pain assessment protocol.

Because this publication reports preliminary results and follow-up data are not yet complete, only preoperative data are included in the present analysis. The full study protocol will be expanded within the PNTR framework, and the resulting data will be reported during the course of the study.

### 2.2. Statistical Analysis

All study data were analyzed descriptively. Categorical variables are presented as absolute frequencies and percentages, and continuous variables as mean, median, quartiles, and range. Because subgroup sizes were limited, exploratory hypothesis testing and correlation analyses were performed using nonparametric methods. Spearman’s rank correlation was used for correlation analysis, and the Mann–Whitney U test or the Kruskal–Wallis test for the comparison of median values between two or three groups, respectively. The two-sided, explorative type I error level was set to 5%. All analyses were performed using the R statistical software (version 4.5.0, www.r-project.org).

Given the limited sample size and biological heterogeneity of several histopathological subgroups, particularly Groups 2–4, all subgroup comparisons should be interpreted as exploratory and hypothesis-generating.

## 3. Results

A total of 91 patients were included, comprising 56 males (61.5%) and 35 females (38.9%), with a mean age of 49 years (range: 12–82). Tumors were located in the upper extremity in 39 patients (42.8%), the lower extremity in 50 patients (54.9%), and the trunk in 2 patients (2.2%). Lesions were right-sided in 55 patients (60.4%) and left-sided in 36 patients (39.5%). The most frequently affected nerves were the peroneal nerve (*n* = 15, 16.4%), brachial plexus (*n* = 11, 12%), and lumbosacral plexus (*n* = 10, 10.9%). Mean height and weight were higher in males (180 cm (162–196), 85 kg (50–150)) than in females (164 cm (145–181), 68 kg (38–115)). Radiating pain was reported by 48 patients (52.7%).

### 3.1. General PainDETECT Scores

Most patients (49/91, 53.8%) had PainDETECT scores ≤ 12, indicating predominantly nociceptive pain. Twenty patients (21.9%) had scores ≥ 19, and 22 patients (24.1%) were in the ambiguous range (13–18). Although males were more frequent in the cohort (61.5%), mean PainDETECT scores were similar between males and females (11.8 vs. 12.1). Patients with obesity (BMI > 30 kg/m^2^) had slightly higher mean pain scores (13.0). Most tumors were located in the lower extremity (54.9%), where PainDETECT scores were marginally higher than in the upper extremity group (mean = 12 in both groups, with more patients in the neuropathic range). These results are summarized in Table 1.

### 3.2. Pain Profiles by Tumor Type and Syndromic Peripheral Nerve Tumors

According to histological grouping, benign tumors (Group 1) were most common (69/91, 75.8%) and had a mean PainDETECT score of 11.9 (range 0–29). Malignant tumors (Group 2) included 6/91 (6.5%) patients with the highest mean score (17; range 0–33) and comprised three MPNSTs and three sarcomas. Group 3 included six rare tumor entities (6.5%) with a mean PainDETECT score of 12.1 (range 1–25): perineurioma (*n* = 3; mean 7, range 1–10), hemangioma (*n* = 2, mean 23, range 21–25), and ganglioneuroma (*n* = 1; end score 6). Group 4 (dropout lesions) included 10 patients (10.9%) with a mean score of 9.1 (range 0–21). Group 4 encompassed angioleiomyoma (*n* = 2) and glomus tumors (*n* = 1), which had the highest end scores in this subgroup (12, 19, and 21). Lipoma (*n* = 2, end score 0, 9), hibernoma (*n* = 1, end score 11), angiolipoma (*n* = 1), hidradenoma (*n* = 1, end score 7), and tumor-free soft tissue (*n* = 2, end score 0, 4) were also part of the dropout lesions. Results are summarized in Table 1.

**Figure 1:**
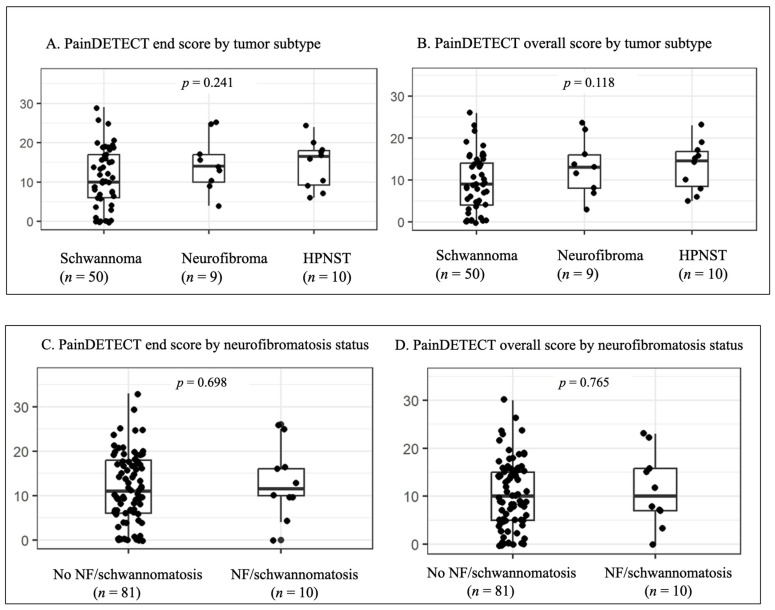
PainDETECT scores by tumor subtype and neurofibromatosis status. Panels (**A**,**B**) show PainDETECT end and overall scores by benign tumor subtype, including schwannoma (*n* = 50), neurofibroma (*n* = 9), and hybrid peripheral nerve sheath tumor (HPNST; *n* = 10). Panels (**C**,**D**) show PainDETECT end and overall scores according to neurofibromatosis/schwannomatosis status. No statistically significant differences were observed between groups.

For subgroup analysis, Group 1 tumors were classified as schwannomas (*n* = 50, 72.4%), Hybrid Peripheral Nerve Sheath Tumors (HPNST; *n* = 10, 14.4%, all hybrid schwannoma/neurofibroma), or neurofibromas (*n* = 9, 13.0%). HPNST and neurofibromas showed numerically higher PainDETECT end and overall scores than schwannomas; however, these differences were not statistically significant (*p* = 0.241 and *p* = 0.118, respectively; Figure 1).

Similar results were observed in patients with a neurofibromatosis spectrum disorder (*n* = 10, 10.9%), including six patients with neurofibromatosis type 1 (NF1), two with LZTR1-related schwannomatosis, and two with non-NF-associated schwannomatosis. PainDETECT end and overall scores did not differ significantly between patients with and without neurofibromatosis-spectrum disorders (*p* = 0.698 and *p* = 0.765, respectively; Figure 1).

Eleven of 91 patients had undergone resection of more than one tumor (12.1%), whereas this information was unknown or not documented in 8 patients (8.8%). Among these 11 patients, the currently analyzed index lesions were schwannomas in 5 cases (45.5%), neurofibromas in 3 cases (27.3%), hybrid nerve sheath tumors in 2 cases (18.2%), and lipoma in 1 case (9.1%). Six patients had undergone surgery for three tumors (54.5%), and two patients had undergone surgery for more than five tumors (18.2%). Seven of the 11 patients with multiple tumor resections had an associated tumor predisposition syndrome (63.6%). In these patients, the PainDETECT questionnaire was completed only for the most recently operated tumor, which was defined as the index lesion. The mean PainDETECT overall score of these index lesions was 11.8, and the mean PainDETECT end score was 13.7.

### 3.3. Relationship Between PainDETECT Scores and Localization

**Figure 2:**
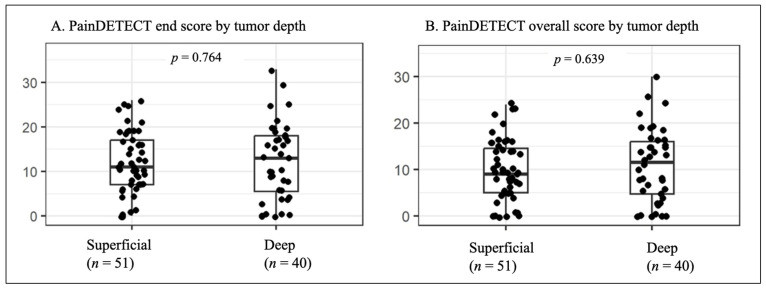
PainDETECT scores by tumor depth. PainDETECT end score (**A**) and overall score (**B**) are shown for superficial tumors (*n* = 51, 56.0%) and deep tumors (*n* = 40, 43.9%). No statistically significant difference was observed between superficial and deep lesions.

To assess the influence of tumor localization on pain, PainDETECT end and overall scores were analyzed according to anatomical localization and the most frequently affected nerves. Tumor depth was classified as superficial in 51 patients (56.0%) and deep in 40 patients (43.9%). Although the initial hypothesis suggested that superficial tumors would be associated with higher PainDETECT scores, the mean scores were slightly higher in deep tumors. This difference was not statistically significant (*p* = 0.764 for end score, *p* = 0.639 for overall score, Figure 2).

**Figure 3:**
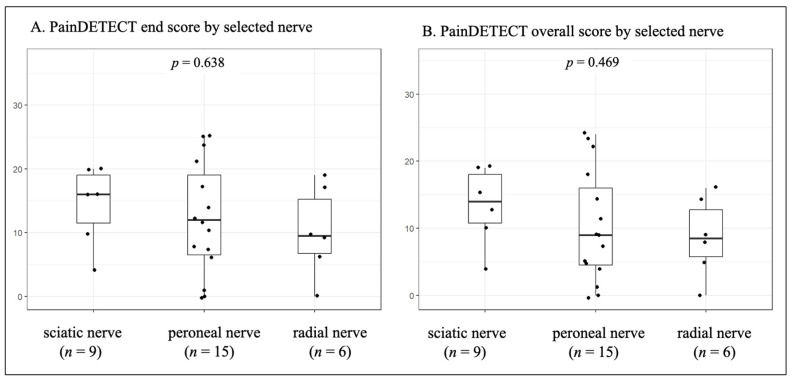
PainDETECT scores by selected peripheral nerves. PainDETECT end score (**A**) and overall score (**B**) are shown for selected anatomically comparable peripheral nerves: sciatic nerve, peroneal nerve, and radial nerve. Plexus lesions were not included in this figure because they represent more complex anatomical structures rather than single peripheral nerves. No statistically significant differences were observed between the visualized nerve subgroups.

Tumors involving the brachial plexus and sciatic nerve tumors had relatively high mean scores (14.6 (range 0–29) and 14.3 (range 4–20), respectively). In contrast, peroneal and lumbosacral plexus lesions had lower mean scores (12.1; range 0–25 and 12.1; range 0–33, respectively). Figure 3 shows PainDETECT end and overall scores for tumors affecting the sciatic, peroneal, and radial nerves. Sciatic nerve tumors showed numerically higher scores; however, these differences were not statistically significant (*p* = 0.638 for the end score, *p* = 0.469 for the overall score).

### 3.4. Relationship Between PainDETECT Scores and Tumor Size

**Figure 4:**
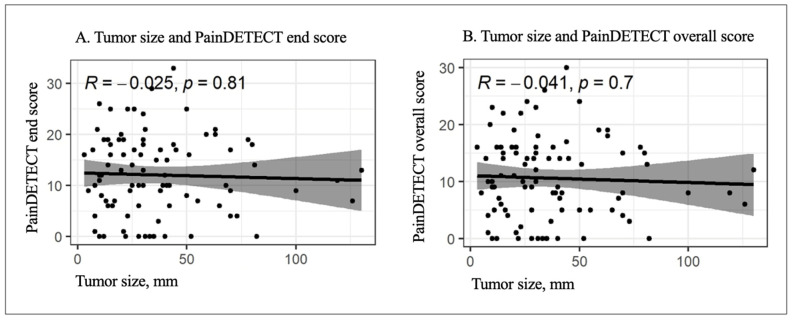
Correlation between tumor size and PainDETECT scores. Scatter plots show the relationship between tumor size and PainDETECT end score (**A**) and overall score (**B**). Spearman’s rank correlation showed no significant association between tumor size and pain scores, with ρ = −0.025, *p* = 0.81 for the end score and ρ = −0.041, *p* = 0.70 for the overall score.

In the overall cohort, tumor size was not significantly correlated with PainDETECT scores (Figure 4). Both the end score (rho = −0.025, *p* = 0.81) and overall score (rho = −0.041, *p* = 0.7) showed very weak, non-significant, negative correlations, indicating no association between tumor size and pain severity in this cohort.

### 3.5. Pain-Course Patterns

**Figure 5:**
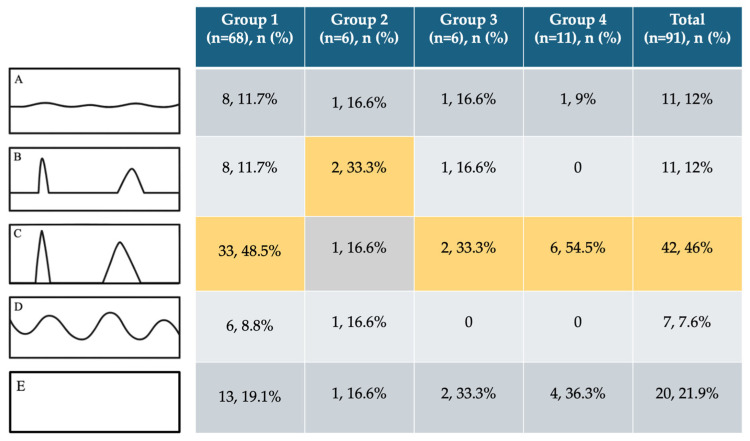
Pain course patterns by histological subgroup. Pain course was assessed using the five predefined PainDETECT patterns: The colored cells indicate values that were highlighted for emphasis; yellow denotes higher relative frequencies compared with the other groups. (**A**), continuous pain with slight fluctuations; (**B**), continuous pain with intermittent pain attacks; (**C**), pain attacks with pain-free intervals; (**D**), pain attacks with residual pain between episodes; and (**E**), no pain. Pattern (**C**) was the most frequent pain course in the overall cohort (*n* = 42, 46%). This was also the most common pain course within each histological subgroup, except in group 2, where type B was most frequent (*n* = 2).

The most common pain-course pattern in the overall cohort was type C (pain attacks with pain-free intervals; Figure 5), observed in 42 of 91 patients (46%). This pattern also predominated in nearly all histological subgroups, except for Group 2, in which type B (continuous pain with intermittent pain attacks) was most frequent (*n* = 2). Pain-free patients (type E) accounted for 21% of the cohort, whereas continuous or fluctuating pain patterns (types A and D) were less common.

### 3.6. Numerical Rating Scale and Pain Dimensions

Pain intensity on the numerical rating scale (NRS, range 0–10; 10 indicating the most severe pain) was assessed as current, maximum, and average pain. Group 2 (malignant tumors) reported the highest pain levels, with a mean current NRS of 4.1, a maximum of 7.1, and an average of 5.5. In contrast, Groups 1 (benign), 3 (rare tumors), and 4 (dropout lesions) had lower mean NRS scores of 3.3, 2.6, and 2.8, respectively.

**Figure 6:**
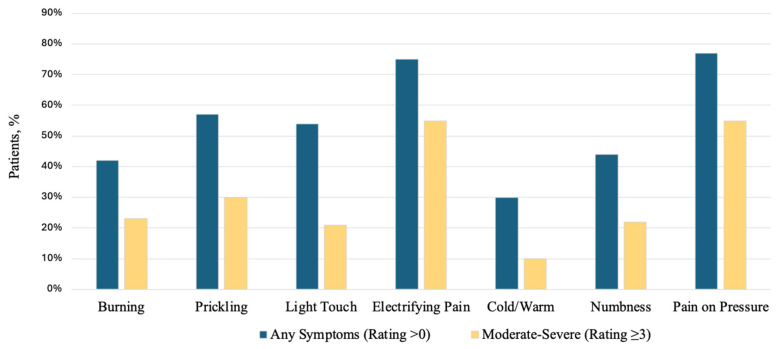
Prevalence and severity of patient-reported pain dimensions (0–5 scale). Bars show the proportion of patients reporting any symptom intensity greater than zero and moderate-to-severe symptom intensity, defined as a rating ≥ 3 on the 0–5 PainDETECT scale. Pressure related and electric-shock-like pain were the most frequent reported symptoms.

Across pain dimensions, the proportion of patients reporting any symptom (rating > 0) ranged from 30% for cold/warm symptoms to 77% for pressure pain. Pressure-related pain (77%) and electric-shock-like pain (75%) were the most frequent symptoms, and both were most often moderate-to-severe (rating ≥ 3; 55% each). Prickling/tingling (57% any symptom; 30% moderate-to-severe) and pain on light touch (54%; 21%) were also common but less frequently moderate-to-severe. Burning pain (42%; 23%) and numbness (44%; 22%) affected a substantial subset of patients, whereas cold/warm symptoms were least prevalent and rarely moderate-to-severe (30%; 10%). These Results are shown in Figure 6. Consistent with these findings, 79 out of 91 patients (86.8%) had a positive Tinel sign on clinical examination.

### 3.7. Comorbidities, Previous Malignancy, and Pain Medication

Data on comorbidities and preoperative medication were available for 73 of 91 patients (80.2%); no data were available for 18 patients (19.8%). Thirty-five patients had no documented relevant comorbidity (35/73, 47.9%). The most frequent comorbidity was arterial hypertension requiring medication (14/73, 19.2%), followed by previous malignancy (8/73, 11.0%), depression (4/73, 5.5%), type 2 diabetes mellitus requiring medication (3/73, 4.1%), and hypercholesterolemia (3/73, 4.1%). A history of malignancy was documented in 8 patients (8/73, 11.0%), including optic glioma in 1 patient (1/73, 1.4%), malignant melanoma in 2 patients (2/73, 2.7%), prostate cancer in 2 patients (2/73, 2.7%), breast cancer in 2 patients (2/73, 2.7%), and testicular cancer in 1 patient (1/73, 1.4%). One patient had previously received chemotherapy with vincristine and carboplatin for optic glioma (1/73, 1.4%).

Preoperative pain medication was not required in most patients with available data (61/73, 83.6%), whereas 12 patients received analgesics (12/73, 16.4%). Medication included non-opioid analgesics, opioid or opioid-like analgesics, and drugs typically used for neuropathic pain, including gabapentin, pregabalin, duloxetine, amitriptyline, or amineurin. Only one patient with a previous malignancy received preoperative pain medication (1/8, 12.5%). Postoperatively, 63 of 73 patients (86.3%) received mainly ibuprofen and/or metamizole as needed for wound pain, which was discontinued according to clinical need.

## 4. Discussion

This study represents one of the first standardized, prospective assessments of preoperative pain symptomatology in syndromic and non-syndromic patients with PNTs using a validated instrument (PainDETECT) in a bicenter setting. The findings highlight pain as a relevant and underrecognized clinical symptom in PNTs, including benign subtypes. Although malignant tumors had the highest mean PainDETECT scores, a substantial proportion of patients with benign tumors, including schwannomas and HPNSTs, also reported moderate to high pain levels.

### 4.1. Malignancy and Syndromic Features

Pain is a common and clinically relevant feature of PNTs and can substantially impair quality of life. Previous studies suggest that approximately three-quarters of patients with a peripheral nerve sheath tumor experience pain in some form [1]. In the present cohort, more than two-thirds of patients reported some degree of pain, whereas only 12% had a pain score of zero.

MPNSTs are aggressive sarcomas that frequently arise from pre-existing plexiform neurofibromas in patients with NF1, although they may occur sporadically [17,18,19]. While MPNSTs are life-threatening malignancies, pain is often the earliest and most prominent clinical symptom. Sudden onset or marked increase in pain intensity, particularly in a previously stable neurofibroma, may indicate malignant transformation. Patients may also present with numbness or focal neurological deficits. Compared with benign peripheral nerve tumors, MPNSTs are typically associated with more severe and persistent pain profiles [20].

The finding that more than 22% of patients had PainDETECT scores ≥ 19, suggesting likely neuropathic pain, supports the concept that pain in PNTs is not limited to malignancy. Even patients with perineurioma (*n* = 3 in this cohort), an entity generally considered less painful [21,22], reported a mean end score of 7 (range, 1–10).

Syndromic patients frequently experience pain. In one PainDETECT-based study, approximately 6 of 20 patients with schwannomatosis scored in the range suggestive of neuropathic pain [23]. Studies comparing painful vs. nonpainful schwannomas have suggested potential molecular mechanisms: painful schwannomas secrete higher levels of certain cytokines and growth factors, such as IL-6, CXCL1 and VEGF, which may sensitize sensory neurons [24]. Systemic medical treatment options have also emerged; for example NF1 patients receiving selumetinib have reported lower pain intensity and reduced pain-related interference with daily activities [6,25]. Pain in non-NF2 schwannomatosis is often chronic, treatment-resistant, and associated with anxiety and depression [26]. In NF1, up to 50–60% of patients with plexiform neurofibromas may experience tumor-associated neuropathic pain [27].

Notably, HPNSTs and neurofibromas showed numerically higher pain scores than schwannomas, although these differences were not statistically significant. HPNSTs remain incompletely understood but are frequently associated with syndromic conditions such as neurofibromatosis and schwannomatosis [28,29].

### 4.2. Size and Location

The hypothesis that superficial tumors cause more pain was not confirmed. Deep lesions showed slightly higher mean scores, although this difference was not statistically significant. Although lower-extremity lesions showed a slightly higher proportion of patients within the neuropathic PainDETECT range, no robust association between anatomical location and specific pain quality was identified in this cohort. Similarly, tumor size was not correlated with pain intensity (rho = −0.025), challenging the assumption that larger tumors invariably generate more pain. Previous studies suggest that pain in PNSTs is heterogeneous and cannot be explained by tumor size or volume alone. Additional factors, including nerve involvement and tumor–nerve interactions, are likely to contribute to pain severity [3,24,30]. Most previous studies have focused on syndromic patients, whereas data in non-syndromic patients remain limited.

The functional profile of the affected nerve is also relevant. In the present cohort, several affected nerves had mixed sensorimotor function, including the sciatic, peroneal, and ulnar nerves, as well as the brachial and lumbosacral plexuses. A strict classification into purely sensory and purely motor nerves was therefore not feasible.

Schwannoma-related pain is often episodic or movement-related rather than spontaneous. Patients may notice pain during specific movements, exercise, or palpation of the lesion, rather than severe nocturnal or resting pain. This pattern is consistent with our finding that pain attacks with pain-free intervals (type C) were common in benign tumors (48.5%) and supports the concept that benign schwannomas may cause pain mainly through nerve compression or stretch.

### 4.3. Clinical Implications

Pain is a major source of morbidity in patients with peripheral nerve tumors, and these prospective baseline data provide clinically relevant insights into the preoperative pain phenotype and patient-reported burden. Because benign tumors represent the largest subgroup, their pain symptoms should not be underestimated but actively addressed in clinical care. Although outcome analyses require complete follow-up, the current findings support timely surgical intervention in selected patients—even those without neurological deficits—when pain is significant, and imaging suggests operability. Future analyses, including follow-up and medication data, will clarify the extent and persistence of pain relief across tumor types. A patient reporting high pain intensity (e.g., NRS 7/10) and PainDETECT scores ≥ 19 may benefit from targeted neuropathic pain treatment. Schwannomas typically cause intermittent, stimulus-dependent pain, often tingling or shooting upon pressure, and usually respond well to surgical removal [10,31]; however, in schwannomatosis, multiple tumors make pain management more complex and frequently require combined medical and surgical strategies. Overall, improved characterization of pain mechanisms in these tumors may support more precise clinical assessment and management.

An additional clinically relevant observation concerns the dropout group. Although these lesions were not histologically confirmed as PNSTs, they had close anatomical nerve contact and clinically mimicked peripheral nerve tumors. Some of these entities, particularly angioleiomyoma and glomus tumors, showed high individual PainDETECT scores. This is consistent with the fact that especially benign subcutaneous tumors may cause severe pain [32,33,34] and therefore frequently lead to surgical resection. Accurate preoperative differentiation from true peripheral nerve tumors remains essential [35].

PainDETECT was selected as an established, standardized, and easy-to-use tool for capturing multiple pain qualities in routine clinical practice. However, elevated scores should be interpreted as neuropathic-like pain features rather than definitive evidence of nerve fiber dysfunction, particularly in benign tumors, where deep somatic pain, pressure effects, or mechanical nerve irritation may also contribute. Treatment decisions should therefore integrate clinical examination, imaging, and the individual pain phenotype.

### 4.4. Influence of Comorbidities, Previous Medical History, Medication, and Multiple Tumors

Comorbidities, previous medical history, and medication use are relevant confounders when interpreting PainDETECT and NRS scores. Data on comorbidities and medication were available for 73 of 91 patients (80.2%). Depression was documented in 4 patients (5.5%), type 2 diabetes mellitus in 3 (4.1%), and previous malignancy in 8 (11.0%). These factors, as well as previous oncological treatment, may influence pain perception and patient-reported outcomes independently of the peripheral nerve tumor itself. Because the subgroups were small and heterogeneous, adjusted statistical analyses were not feasible.

Preoperative pain medication was required in only 12 of 73 patients (16.4%), whereas most patients with available data did not require preoperative analgesics (61/73, 83.6%). Medication included non-opioid analgesics, opioid or opioid-like analgesics, and drugs commonly used for neuropathic pain. These treatments may have influenced PainDE-TECT and NRS scores. Therefore, low pain scores should not automatically be interpreted as evidence of an intrinsically painless lesion, whereas high scores may reflect insufficiently controlled pain, mixed nociceptive–neuropathic pain, or tumor-related nerve irritation. Postoperative medication consisted mainly of ibuprofen and/or metamizole as needed for wound pain in 63 of 73 patients (86.3%) and was therefore not interpreted as a standardized postoperative pain outcome.

Patients with multiple tumors represent an important subgroup, particularly in syndrome-associated peripheral nerve tumors. In this cohort, 11 of 91 patients had undergone more than one tumor resection (12.1%), and 7 of these 11 patients had an associated tumor predisposition syndrome (63.6%). In this interim analysis, PainDETECT was assessed prospectively only for the most recently operated index lesion, while previous tumor resections were documented retrospectively.

Future PNTR analyses will assess PainDETECT before each new tumor operation and separately for each tumor, aiming to identify which tumors, locations, or histological subtypes are associated with the highest pain burden in patients with multiple lesions. The final PNTR analysis is planned once at least 200 patients with histologically confirmed peripheral nerve tumors have been included, excluding the drop-out cohort, and complete follow-up data are available.

### 4.5. Limitations and Strengths

Limitations: This study has several limitations. First, the cohort predominantly comprised benign tumors, limiting generalizability to malignant and rare entities. Second, subgroup sizes were small; therefore, all histopathological comparisons should be interpreted as exploratory and hypothesis-generating. Third, postoperative follow-up data were incomplete, precluding analysis of longitudinal pain trajectories and postoperative neurological deficits. In the present analysis, postoperative deficits were not evaluated because the primary focus was on preoperative pain symptomatology. In the overall PNTR cohort, malignant tumors appear to be associated with more frequent preoperative pain, often accompanied by motor and sensory deficits that may persist; these data are not included in the present publication and will be analyzed separately. Fourth, comorbidities and medication use represent relevant confounders. Depression, diabetes mellitus, previous malignancy, prior chemo- or radiotherapy, chronic pain disorders, neuropathic pain conditions, and analgesic or neuropathic pain medication may influence PainDETECT and NRS scores independently of the tumor itself. Although these variables were reviewed descriptively, incomplete documentation and the limited sample size prevented adjusted analyses. Therefore, tumors should not be classified as intrinsically painful or painless based solely on PainDETECT scores. Finally, the lesion–nerve relationship, including extrinsic compression, displacement, stretching, infiltration, or destruction, could not be reliably classified in all cases.

Strengths: This study is among the first standardized, prospective assessments of preoperative pain symptomatology in patients with peripheral nerve tumors using a validated instrument (PainDETECT) in a bicenter setting. Its focus on pain, often the dominant patient-reported burden, adds substantial clinical relevance.

## 5. Conclusions

Pain is a common symptom in patients with peripheral nerve tumors, including those with benign histology. Malignant tumors showed the highest pain scores, but benign and hybrid lesions were also frequently associated with clinically relevant symptoms. Pain intensity was not correlated with tumor size, depth, or nerve localization. Electric-shock-like and pressure-related sensations were predominant, reflected by the high rate of positive Tinel signs. These findings underscore the clinical relevance of structured pain assessment across all tumor subtypes. The goal is to expand this assessment to a multicenter setting within the PNTR framework.

## Figures and Tables

**Table 1:** Demographic and clinical characteristics and their association with the PainDETECT end score in the total cohort.

painDETECT Score	≤12 *n*	13–18 *n*	≥19 *n*	Mean (Range)	Lowest Score (0), *n*
Patients, *n* = 91 (100%)	49	22	20	11.9 (0–33)	11
Male, *n* = 56 (61.5%)	30	11	14	11.8 (0–29)	7
Female, *n* = 35 (38.9%)	18	11	6	12.1 (0–33)	4
BMI (kg/m^2^) *					
<18.5 *n* = 1 (1%)	0	1	0	17.0 (17)	0
18.5–24.9 *n* = 45 (49.4%)	27	7	11	12.0 (0–33)	5
25–29.9 *n* = 30 (32.9%)	16	8	6	10.7 (0–25)	5
>30 *n* = 13 (14.2%)	6	5	2	13.0 (0–29)	1
Location					
UE, *n* = 39 (42.8%)	21	11	7	11.8 (0–29)	4
LE, *n* = 50 (54.9%)	27	11	12	11.9 (0–29)	6
Trunk, *n* = 2 (2.1%)	1	0	1	13.0 (0, 26)	1
Groups					
1 (Benign), *n* = 69 (75.8%)	36	19	14	11.9 (0–29)	8
2 (Malignant), *n* = 6 (6.5%)	1	3	2	16.8 (0–33)	1
3 (Rare), *n* = 6 (6.5%)	4	0	2	12.1 (1–25)	0
4 (Dropouts), *n* = 10 (10.9%)	8	0	2	9.1 (0–21)	2
Most effected Nerves:					
Peroneal Nerve, *n* = 15 (16.4%)	9	2	4	12.1 (0–25)	2
Brachial Plexus, *n* = 11 (12.0%)	4	3	4	14.6 (0–29)	2
LS Plexus, *n* = 10 (10.9%)	5	3	2	12.1 (0–33)	1
Ulnar Nerve, *n* = 9 (9.8%)	3	2	2	10.5 (0–25)	1
Sciatic Nerve, *n* = 6 (6.5%)	2	2	2	14.3 (4–20)	0
Tumor depth					
Superficial, *n* = 51 (56.0%)	30	10	11	11.6 (0–26)	5
Deep, *n* = 40 (43.9%)	19	12	9	12.3 (0–33)	6

* Missing *n* = 2, UE = Upper Extremity, LE = Lower Extremity, LS = Lumbosacral.

## Data Availability

The authors will make the raw data supporting this article’s conclusion available upon request.

## Abbreviations

EQ-5D-5L	Euro-QoL-5D-5L
EQ-VAS	Euro-QoL visual analogue scale
F	Female
HPNST	Hybrid Peripheral Nerve Sheath Tumor
M	Male
MPNST	Malignant Peripheral Nerve Sheath Tumor
MRI	Magnetic Resonance Imaging
NF1	Neurofibromatosis 1
NRS	Numerical Rating Scale
LE	Lower Extremity
PNTR	Peripheral nerve tumor registry
PNT	Peripheral Nerve Tumor
PSQI	Pittsburgh Sleep Quality Index
UE	Upper Extremity

## References

[1] Sughrue M.E., Levine J., Barbaro N.M. (2008). Pain as a Symptom of Peripheral Nerve Sheath Tumors: Clinical Significance and Future Therapeutic Directions. J. Brachial Plex. Peripher. Nerve Inj..

[2] Uerschels A.K., Dengler N.F., Chihi M., Lenkeit A., Dinger T.F., Jabbarli R., Sure U., Hagenacker T., Wrede K.H., Gembruch O. (2023). Benign Peripheral Nerve Sheath Tumors: An Interdisciplinary Diagnostic and Therapeutic Challenge. Neurosurg. Rev..

[3] Zhou H., Yao C., Dong Y., Alhaskawi A., Wang Z., Lai J., Ezzi S.H.A., Kota V.G., Abdulla M.H.A.H., Lu H. (2022). Clinical Characteristics and Management Experience of Schwannoma in Extremities: Lessons Learned from a 10-Year Retrospective Study. Front. Neurol..

[4] Merker V.L., Esparza S., Smith M.J., Stemmer-Rachamimov A., Plotkin S.R. (2012). Clinical Features of Schwannomatosis: A Retrospective Analysis of 87 Patients. Oncologist.

[5] Rubright R., Caterina M.J., Belzberg A., Ostrow K.L. (2025). Conditioned Medium from Painful Non-NF2 Schwannomatosis Tumors Increases Pain Behaviors in Mice. Sci. Rep..

[6] Gui C., Canthiya L., Zadeh G., Suppiah S. (2024). Current State of Spinal Nerve Sheath Tumor Management and Future Advances. Neurooncol. Adv..

[7] Kim D.H., Murovic J.A., Tiel R.L., Kline D.G. (2004). Operative Outcomes of 546 Louisiana State University Health Sciences Center Peripheral Nerve Tumors. Neurosurg. Clin. N. Am..

[8] Knight S.W.E., Knight T.E., Santiago T., Murphy A.J., Abdelhafeez A.H. (2022). Malignant Peripheral Nerve Sheath Tumors—A Comprehensive Review of Pathophysiology, Diagnosis, and Multidisciplinary Management. Children.

[9] Da J.L.W., Merker V.L., Jordan J.T., Ly K.I., Muzikansky A., Parsons M., Wolters P.L., Xu L., Styren S., Brown M.T. (2022). Design of a Randomized, Placebo-Controlled, Phase 2 Study Evaluating the Safety and Efficacy of Tanezumab for Treatment of Schwannomatosis-Related Pain. Contemp. Clin. Trials.

[10] Grübel N., Antoniadis G., Ak U., Mayer B., König R., Wirtz C.R., Pala A., Dengler N.F., Pedro M.T. (2024). Health-Related Quality of Life in Patients with Peripheral Nerve Tumors: Results from the German Multicentric Peripheral Nerve Tumor Registry. Front. Oncol..

[11] El Sayed L., Masmejean E.H., Lavollé A., Biau D., Peyre M. (2022). Clinical Results after Surgical Resection of Benign Solitary Schwannomas: A Review of 150 Cases. Orthop. Traumatol. Surg. Res..

[12] Dengler N.F., Scholz C., Beck J., Uerschels A.K., Sure U., Scheller C., Strauss C., Martin D., Schackert G., Heinen C. (2023). Rationale and Design of the Peripheral Nerve Tumor Registry: An Observational Cohort Study. Neurol. Res..

[13] Freynhagen R., Baron R., Gockel U., Tölle T.R. (2006). PainDETECT: A New Screening Questionnaire to Identify Neuropathic Components in Patients with Back Pain. Curr. Med. Res. Opin..

[14] Cappelleri J.C., Bienen E.J., Koduru V., Sadosky A. (2014). Measurement Properties of PainDETECT by Average Pain Severity. Clin. Outcomes Res..

[15] Grochtdreis T., Dams J., König H.H., Konnopka A. (2019). Health-Related Quality of Life Measured with the EQ-5D-5L: Estimation of Normative Index Values Based on a Representative German Population Sample and Value Set. Eur. J. Health Econ..

[16] Mollayeva T., Thurairajah P., Burton K., Mollayeva S., Shapiro C.M., Colantonio A. (2016). The Pittsburgh Sleep Quality Index as a Screening Tool for Sleep Dysfunction in Clinical and Non-Clinical Samples: A Systematic Review and Meta-Analysis. Sleep Med. Rev..

[17] Evans D.G.R., Baser M.E., McGaughran J., Sharif S., Howard E., Moran A. (2002). Malignant Peripheral Nerve Sheath Tumours in Neurofibromatosis 1. J. Med. Genet..

[18] Somatilaka B.N., Sadek A., McKay R.M., Le L.Q. (2022). Malignant Peripheral Nerve Sheath Tumor: Models, Biology, and Translation. Oncogene.

[19] Wanebo J.E., Malik J.M., Vandenberg S.R., Wanebo H.J., Driesen N., Persing J.A. (1993). Malignant Peripheral Nerve Sheath Tumors. A Clinicopathologic Study of 28 Cases. Cancer.

[20] Yao C., Zhou H., Dong Y., Alhaskawi A., Hasan Abdullah Ezzi S., Wang Z., Lai J., Goutham Kota V., Hasan Abdulla Hasan Abdulla M., Lu H. (2023). Malignant Peripheral Nerve Sheath Tumors: Latest Concepts in Disease Pathogenesis and Clinical Management. Cancers.

[21] Brand C., Pedro M.T., Pala A., Heinen C., Scheuerle A., Braun M., Antoniadis G. (2021). Perineurioma: A Rare Entity of Peripheral Nerve Sheath Tumors. J. Neurol. Surg. A Cent. Eur. Neurosurg..

[22] Uerschels A.K., Krogias C., Junker A., Sure U., Wrede K.H., Gembruch O. (2020). Modern Treatment of Perineuriomas: A Case-Series and Systematic Review. BMC Neurol..

[23] Farschtschi S.C., Mainka T., Glatzel M., Hannekum A.L., Hauck M., Gelderblom M., Hagel C., Friedrich R.E., Schuhmann M.U., Schulz A. (2020). C-Fiber Loss as a Possible Cause of Neuropathic Pain in Schwannomatosis. Int. J. Mol. Sci..

[24] Ostrow K.L., Donaldson K.J., Caterina M.J., Belzberg A., Hoke A. (2019). The Secretomes of Painful Versus Nonpainful Human Schwannomatosis Tumor Cells Differentially Influence Sensory Neuron Gene Expression and Sensitivity. Sci. Rep..

[25] Chen A.P., Coyne G.O.S., Wolters P.L., Martin S., Farschtschi S., Blanco I., Chen Z., Darrigo L.G., Eoli M., Whittle J.R. (2025). Efficacy and Safety of Selumetinib in Adults with Neurofibromatosis Type 1 and Symptomatic, Inoperable Plexiform Neurofibromas (KOMET): A Multicentre, International, Randomised, Placebo-Controlled, Parallel, Double-Blind, Phase 3 Study. Lancet.

[26] Hino U., Tamura R., Toda M. (2025). Optimal Delivery of Pain Management in Schwannomatosis: A Literature Review. Ther. Clin. Risk Manag..

[27] Miele G., Russo I., Filipponi L., Perrotta S., Maida E., Piluso G., Melone M.A.B., Santoro C. (2025). Clinical Efficacy of Selumetinib in Alleviating Neuropathic Pain Associated with Plexiform Neurofibroma: A Case Series. Genes.

[28] Lenartowicz K.A., Monie D.D., Amrami K.K., Klein C.J., Giannini C., Spinner R.J. (2023). Hybrid Tumors with Perineurioma Components: A Systematic Review of the Literature and Illustrative Case. Acta Neurochir..

[29] Salzano S., Caltabiano R., Zanelli M., Palicelli A., Zizzo M., Koufopoulos N., Boutas I., Magro G., Barresi V., Broggi G. (2025). Hybrid Benign Peripheral Nerve Sheath Tumors: A Comprehensive Literature Review with Emphasis on Their Clinical, Morphological and Genetic Features. Diagnostics.

[30] Gonzalvo A., Fowler A., Cook R.J., Little N.S., Wheeler H., McDonald K.L., Biggs M.T. (2011). Schwannomatosis, Sporadic Schwannomatosis, and Familial Schwannomatosis: A Surgical Series with Long-Term Follow-up: Clinical Article. J. Neurosurg..

[31] Istefan E., Zimmerman M., Dahlin L.B., Nyman E. (2023). Benign Nerve Tumours in the Upper Limb: A Registry-Based Study of Symptoms and Surgical Outcome. Sci. Rep..

[32] Assmus H., Dombert T. (2002). Zur Lokalisation Und Operativen Behandlung Der Glomustumoren Der Extremitäten. Bericht Über 36 Fälle. Handchir. Mikrochir. Plast. Chir..

[33] Burket J.M., Trask D.M. (1993). Painful Tumors of the Skin: “LEND AN EGG”. J. Am. Acad. Dermatol..

[34] Wang Y., Li T., Lv Z., Bian Y., Feng B., Liu Y., Zhou X., Weng X. (2022). Glomus Tumors around or in the Knee: A Case Report and Literature Review. BMC Surg..

[35] Grübel N., Pedro M.T., Antoniadis G., Durner G., Wirtz C.R., Pöschl P., Dengler N., Wrede K., Gembruch O., Uerschels A.K. (2026). Beyond Nerve Tumors: Differential Diagnoses in Suspected Peripheral Nerve Sheath Tumors. Brain Spine.

